# Identification of New Alleles and the Determination of Alleles and Genotypes Frequencies at the *CYP2D6* Gene in Emiratis

**DOI:** 10.1371/journal.pone.0028943

**Published:** 2011-12-22

**Authors:** Rula Y. Qumsieh, Bassam R. Ali, Yousef M. Abdulrazzaq, Ossama Osman, Nadia A. Akawi, Salim M. A. Bastaki

**Affiliations:** 1 Department of Pharmacology, Faculty of Medicine and Health Sciences, United Arab Emirates University, Al-Ain, United Arab Emirates; 2 Department of Pathology, Faculty of Medicine and Health Sciences, United Arab Emirates University, Al-Ain, United Arab Emirates; 3 Department of Pediatrics, Faculty of Medicine and Health Sciences, United Arab Emirates University, Al-Ain, United Arab Emirates; 4 Department of Psychiatry, Faculty of Medicine and Health Sciences, United Arab Emirates University, Al-Ain, United Arab Emirates; Dr. Margarete Fischer-Bosch Institute of Clinical Pharmacology, Germany

## Abstract

CYP2D6 belongs to the cytochrome P450 superfamily of enzymes and plays an important role in the metabolism of 20–25% of clinically used drugs including antidepressants. It displays inter-individual and inter-ethnic variability in activity ranging from complete absence to excessive activity which causes adverse drug reactions and toxicity or therapy failure even at normal drug doses. This variability is due to genetic polymorphisms which form poor, intermediate, extensive or ultrarapid metaboliser phenotypes. This study aimed to determine *CYP2D6* alleles and their frequencies in the United Arab Emirates (UAE) local population. *CYP2D6* alleles and genotypes were determined by direct DNA sequencing in 151 Emiratis with the majority being psychiatric patients on antidepressants. Several new alleles have been identified and in total we identified seventeen alleles and 49 genotypes. CYP2D6*1 (wild type) and CYP2D6*2 alleles (extensive metaboliser phenotype) were found with frequencies of 39.1% and 12.2%, respectively. CYP2D6*41 (intermediate metaboliser) occurred in 15.2%. Homozygous CYP2D6*4 allele (poor metaboliser) was found with a frequency of 2% while homozygous and heterozygous CYP2D6*4 occurred with a frequency of 9%. CYP2D6*2xn, caused by gene duplication (ultrarapid metaboliser) had a frequency of 4.3%. *CYP2D6* gene duplication/multiduplication occurred in 16% but only 11.2% who carried more than 2 active functional alleles were considered ultrarapid metabolisers. *CYP2D6* gene deletion in one copy occurred in 7.5% of the study group. In conclusion, *CYP2D6* gene locus is heterogeneous in the UAE national population and no significant differences have been identified between the psychiatric patients and controls.

## Introduction

Interethnic variation in the capacity to metabolize drugs is mainly due to genetic constitution [1]. Recent advances in pharmacogenomics elucidated that some variations in DNA sequences recognized as single nucleotide polymorphisms (SNPs) may explain some of the variability in drug metabolizing enzyme activities. These contribute to drug-induced adverse reactions, toxicity, and therapeutic responses in different ethnic groups [2]. The best characterized genetically determined variations in antidepressant drug metabolism are those associated with the polymorphic N-acetyltransferase, NAT2, and two polymorphic cytochrome P450s involved with oxidation reactions, CYP2D6 and CYP2C19, all of which show marked interethnic differences in catalytic activity and allele distribution [3]. Whereas the clinical significance of the CYP2C19 polymorphism is uncertain, the clinical consequences of the CYP2D6 polymorphism are well established [4], [5].

The potential importance of interethnic differences in *CYP2D6* gene structure and expression lies in the large number of drugs whose elimination is catalysed by CYP2D6. These include tricyclic antidepressants and antipsychotics [6]. The *CYP2D6* locus is highly polymorphic and currently more than 120 variant *CYP2D6* alleles have been described (www.cypalleles.ki.se/cyp2d6.htm). These alleles can be divided into; 1) alleles resulting in no functional product (poor metabolizers, PMs); 2) alleles causing a reduced rate of metabolism (intermediate metabolizers, IMs); 3) alleles causing ultrarapid metabolism (ultra-rapid metabolizers, UMs); and 4) alleles with no important functional consequences (extensive metabolizers, EMs) [7] Given the relatively wide spread use of CYP2D6-metabolised medications, including antidepressants, and limited availability of data regarding the alleles and genotypes in Emiratis, this study was undertaken to determine *CYP2D6* alleles and their frequencies, using direct DNA sequencing of the full coding regions and large parts of the intronic sequences, in this population.

## Results

### Identification of new *CYP2D6* alleles

The DNA sequence analysis of all the coding regions, the splice sites and large parts of the intronic regions was carried out for 151 subjects from the UAE local population. This included 50 healthy controls and 101 subjects on antidepressants. Eight out of 151 subjects had new alleles and new genotypes (Table 1 and 2). CYP2D6*105 was the only new homozygous genotype where we found a novel mutation c.1097T>C (g.3268T>C) which causes p.F366S change in the corresponding protein. This mutation is accompanied by the two common SNPs, namely p.R296C and p.S486T (http://www.cypalleles.ki.se/cyp2d6.htm) in a homozygous state. The other new alleles were unique in their combinations. Five patients were carrying unreported combinations of known SNPs with some unknowns (Table 2). Both new SNPs g.1783 A>C and g.4028C>A were intronic thus their effect on the enzyme function is not clear. The ascertained haplotypes in these subjects were undetermined due to the heterozygosity of some of their SNPs. Parents were not included in this study and therefore it was not possible to assign the alleles.

**Table 1:** New alleles found in *CYP2D6* among our UAE study group.

New Alleles	DNA change¥	Protein
CYP2D6*102	Intron 1 conversion with CYP2D7 (214–245); 310G>T; **972C>T**; 1661G>C; **2850C>T**; 3384A>C; 3790C>T; **4180G>C**; 4481G>A	A90V; R296C; S486T
CYO2D6*103	Intron 1 conversion with CYP2D7 (214–245); 310G>T;**972C>T**;1661G>C;**1749A>G**;**2850C>T**; 3384A>C; 3790C>T; **4180G>C**; 4481G>A	A90V; N166D; R296C; S486T
CYP2D6*104	Intron 1 conversion with CYP2D7 (214–245); 310G>T; 843T>G; 1661G>C; **1720A>T**; **2850C>T**; 3384A>C; 3790C>T; **4180G>C**; 4481G>A	E156A; R296C; S486T
CYP2D6*105	Intron 1 conversion with CYP2D7 (214–245); 310G>T; 746C>G; 843T>G; 1661G>C; **2850C>T**; **3268T>C**; 3384A>C; 3790C>T; **4180G>C**; 4481G>A	R296C; **F366S**; S486T

Nucleotide variations in **bold** are the major SNPs/alterations responsible for the phenotype of the corresponding allele.

Nucleotide and amino acid variations underlined are novel and have not been reported previously in the *CYP2D6* allele nomenclature database.

¥ All nucleotide changes are based on Genbank accession number M33388* following the instructions in database http://www.cypalleles.ki.se/cyp2d6.htm.

**Table 2:** New genotypes found in *CYP2D6* among our UAE study group.

New Genotypes	DNA change¥	Protein change
Genotype 1	Homozygous SNPs: Intron 1 conversion with CYP2D7 (214–245); 310G>T; 843T>G; 1661G>C; **2850C>T**; 3384A>C; 3790C>T; **4180G>C**. Heterozygous SNPs: **984A>G/A**; 2661G>A/G; 4028C>A/C	H94R; R296C; S486T
Genotype 2	Homozygous SNPs:310G>T. Heterozygous SNPs **2606G>A/G**; **2610T>A/T**	E278K; M279K
Genotype 3	Homozygous SNPs: 310G>T; 1661G>C; **3183G>A**; 3384A>C; **4180G>C**. Heterozygous SNPs: **100C>T/C**; 1039C>T/C; **2850C>T/C**; 4401C>T/C	P34S; R296C; V338M; S486T
Genotype 4	Homozygous SNPs: 310G>T; 1661G>C; **2850C>T**; 3384A>C; 3790C>T; **4180G>C**; 4481G>A. Heterozygous SNPs: Intron 1 conversion with CYP2D7(214–245);**1749A>G/A**; 1783A>C/A; **2988G>A/G**	N166D; R296C; Splicing defect; S486T
Genotype 5	Homozygous SNPs: 4481G>AHeterozygous SNPs: Intron 1 conversion with CYP2D7 (214–245); 310G>T/G; 843T>G/T; 1661G>C/G; **1749 A>C/A**; 1783 A>C/A; **2850C>T/C**; 3384A>C/A; 3790C>T/C; **4180G>C/G**	N166D; R296C; S486T

Nucleotide variations in **bold** are the major SNPs/alterations responsible for the phenotype of the corresponding allele.

Nucleotide and amino acid variations underlined are not reported in *CYP2D6* allele nomenclature database.

¥ All nucleotide change is based on Gene M33388* following the databasehttp://www.cypalleles.ki.se/cyp2d6.htm.

### *CYP2D6* allele frequencies among 151 Emiratis

Within our sequenced study group (*n* = 151, 302 alleles) 17 different alleles were identified in which CYP2D6*1 occurred in the highest frequency of 39.1% (*n* = 118) (Table 3). This was followed by CYP2D6*41 with a frequency of 15.2% (*n* = 46), while CYP2D6*2, CYP2D6*4, CYP2D6*2xn, CYP2D6*39 and CYP2D6*10 had frequencies of 12.2%, 9%, 4.3%, 4% and 3.3%, respectively.

**Table 3:** *CYP2D6* allele frequencies among 151 (302 alleles) UAE nationals.

Alleles presence as homozygous or heterozygous	Allele frequency %. N = 151. 302 alleles total
CYP2D6*1	39.1
CYP2D6*41	15.2
CYP2D6*2	12.2
CYP2D6*4	9
CYP2D6*2xn	4.3
CYP2D6*39	4
CYP2D6*10	3.3
CYP2D6*New	2.6
CYP2D6*35	2
CYP2D6*17	2.5
CYP2D6*29	1.60
CYP2D6*1xn	1.60
CYP2D6*34	1
CYP2D6*27	0.7
CYP2D6*10xn	0.3
CYP2D6*43	0.3
CYP2D6*46	0.3
*Total*	100%

### Frequencies of *CYP2D6* functional alleles among 151 UAE individuals

As shown in table 4 there were only 3 (2%) individuals out of 151 who carried no functional alleles at all. Of the 151 subjects, 27 (17.9%) had only one active allele. The majority carried two active alleles with a frequency of 68.9%, whereas 17 (11.3%) individuals were carrying more than two active alleles.

**Table 4:** The number of active alleles in the sample studied (*n = 151*).

Number of active alleles	N	%
0 active alleles	3	2
1 active allele	27	17.9
2 active alleles	104	68.9
>2 active alleles	17	11.3

### Frequency of *CYP2D6* Genotypes and their predicted phenotypes

CYP2D6*1, CYP2D6*2 and CYP2D6*39 encode for EMs phenotype in both homozygous and heterozygous forms. CYP2D6*10 and CYP2D6*41 encode for IMs phenotype but only in the homozygous form or when it is combined with one of the poor metaboliser alleles such as CYP2D6*4 which only encode for a PM phenotype in the homozygous form [8]–[10]. CYP2D6*2xn which indicates the presence of gene duplication encode for a UMs phenotype [11].

In addition to the new genotypes described in table 2, we found 44 different already known genotypes among our study group (Table 5). Twenty three out of the 44 genotypes were assigned under EMs phenotype (homozygous or heterozygous). Twelve out of the 44 were genotypes associated with duplication or multiduplication in one of their alleles and they were assigned under UMs phenotype. Eight out of 44 genotypes were assigned as IMs phenotype. One genotype was predicted to have PM phenotype.

**Table 5:** The observed distribution of *CYP2D6* known genotypes and their predicted phenotypes in the recruited individuals (*n* = 151).

*#*	*CYP2D6* genotype	Predicted phenotype	N	%	No of active alleles
**1**	*1/*1(wild type)	Homozygous EM	31	20.5	2
**2**	*1/*1xn	Homozygous UM	1	0.7	3
**3**	*1/*2	Heterozygous EM	17	11.3	2
**4**	*1/*2xn	Heterozygous UM	5	3.3	3
**5**	*2/*2	Homozygous EM	3	2	2
**6**	*2xn/*2	Homozygous UM	1	0.7	3
**7**	*2/*4	Heterozygous EM	3	2	1
**8**	*2xn/*4	Heterozygous EM	2	1.3	2
**9**	*2/*41	Heterozygous EM	2	1.3	2
**10**	*2xn/*41	Heterozygous UM	1	0.7	3
**11**	*2/*39	Heterozygous EM	4	2.6	2
**12**	*2xn/*39	Heterozygous UM	2	1.3	3
**13**	*2xn/*10	Heterozygous UM	1	0.7	3
**14**	*2xn/*17	Heterozygous UM	1	0.7	3
**15**	*2/*29	Heterozygous EM	1	0.7	2
**16**	*1/*41	Heterozygous EM	14	9.3	2
**17**	*1xn/*41	Heterozygous UM	1	0.7	3
**18**	*1/*4	Heterozygous EM	8	5.3	1
**19**	*1/*35	Heterozygous EM	1	0.7	2
**20**	*1/10	Heterozygous EM	2	1.3	2
**21**	*1xn/10	Heterozygous UM	1	0.7	3
**22**	*1/*34	Heterozygous EM	3	2	2
**23**	*1/*17	Heterozygous EM	1	0.7	2
**24**	*1/*27	Heterozygous EM	1	0.7	2
**25**	*1xn/*27	Heterozygous UM	1	0.7	3
**26**	*1/*29	Heterozygous EM	1	0.7	2
**27**	*1xn/*39	Heterozygous UM	1	0.7	4
**28**	*1/*46	Heterozygous EM	1	0.7	2
**29**	*4/*4	Homozygous PM	3	2	0
**30**	*4/*41	Heterozygous IM	6	4	1
**31**	*4/*29	Heterozygous IM	1	0.7	1
**32**	*4/*43	Heterozygous EM	1	0.7	1
**33**	*41/*41	Homozygous IM	8	5.3	2
**34**	*35/*35	Homozygous EM	1	0.7	2
**35**	*10/*10	Homozygous IM	1	0.7	2
**36**	*29/*29	Homozygous IM	1	0.7	2
**37**	*17/*17	Homozygous IM	1	0.7	2
**38**	*17/*41	Heterozygous IM	1	0.7	2
**39**	*17/*39	Heterozygous EM	1	0.7	2
**40**	*10/*41	Heterozygous IM	1	0.7	2
**41**	*10xn/*41	Heterozygous UM	1	0.7	3
**42**	*10/*35	Heterozygous EM	1	0.7	2
**43**	*35/*39	Heterozygous EM	2	1.3	2
**44**	*39/*41	Heterozygous EM	2	1.3	2

*EM* extensive metabolizer, *PM* poor metabolizer, *UM* ultrarapid metabolizer and *IM* intermediate metabolizer.

In the subjects studied (*n* = 151) the following *CYP2D6* genotypes were identified with the highest occurrence:*1/*1(*n* = 31),*1/*2 (*n* = 17),*1/*41(*n* = 14),*1/*4(*n* = 8), *41/*41(*n* = 8), *4/*41 (*n* = 6), *2/*39 (*n* = 4) and *1/*2xn (*n* = 5) (Table 5). Out of the 151 study group 2% (*n* = 3) were poor metabolizers carrying the *4/*4 genotype.

## Discussion

The impact of CYP2D6 polymorphisms on the clinical outcome of psychoactive drugs has been extensively described in the literature [12]. CYP2D6 has more than 120 allelic variants resulting from point mutations, rearrangements, additions, deletions and duplications. Several studies have shown that the frequencies of the alternative CYP2D6 phenotypes vary significantly among different ethnic groups [2], [13], [14]. Examples of these ethnically specific markers are CYP2D6*17, found in Africans, and CYP2D6*10, found in Asians. Both result in slower metabolism of CYP2D6 substrates (such as risperidone and paroxetine), which result in higher plasma concentrations thereby needing lower doses for therapeutic effect [8], [12], [15], [16].

Duplications or multiplications of the *CYP2D6* gene occurs in 1% of Swedes, 5% of Spanish, 29% of Ethiopians and 19% of Arabs results in an increased number of enzymes available for metabolic processes [3], [7], [17]. This is called ultrarapid metabolization. Patients with these mutations will require higher doses of medications to achieve the therapeutic response. UMs were previously regarded as “noncompliant” because they did not respond to the standard medication doses administered [18]. *CYP2D6* genotypic frequency information had been determined for most populations around the world [12]. They displayed significant interethnic differences in *CYP2D6* allele frequencies [1] resulting in variable percentages of PMs, IMs, EMs and UMs in a given population. This is the first study to genotype *CYP2D6* gene in UAE local population. However, the incidence of different *CYP2D6* polymorphisms in the UAE population holds a great challenge to be characterized because of the huge ethnic diversity that exists in the country.

**Figure 1:**
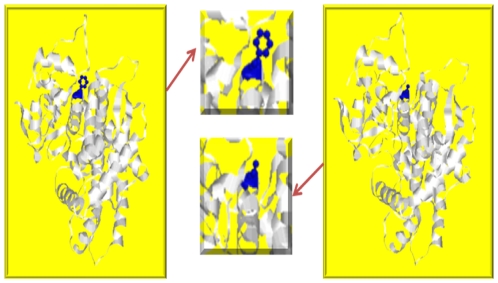
CYP2D6 protein showing position 366 where there is a substitution from phenylalanine to serine amino acid. Molecular modeling was performed using RasMol (http://www.openrasmol.org). A) 366 Phenylalanine is large hydrophobic amino acid B) 366 Serine is a polar uncharged small Amino acid.

The novel variant p.F366S found in this population has not been reported elsewhere and its clinical impact is unknown, but theoretically and based on bioinformatics tools it could be clinically significant. It changed phenylalanine which is a hydrophobic large amino acid into serine which is a polar uncharged small amino acid. The phenyl ring on the phenylalanine probably interacts with the beta sheet and changes it into small hydrophilic molecule thus disrupting the reaction and the structure (Figure 1). Moreover, this amino acid substitution is predicted to probably be protein damaging based on analysis using PolyPhen program for prediction of functional effect of human SNPs (http://genetics.bwh.harvard.edu/pph/). Unfortunately, no plasma was available for the homozygous subject, thus we were unable to determine his metabolic ratio (MR).

*CYP2D6* allele frequencies in our UAE study group were compared with frequencies seen in other ethnic populations. According to Toscano et al. [19] *1*,*2 and*41 are the three most frequent *CYP2D6* functional alleles. This is similar to our findings as the said alleles occurred in the highest frequencies namely: *1 (39.1%), *41 (15.2%) and *2 (12.2%). Unlike other populations [19], CYP2D6*41 occurred more frequently than CYP2D6*2 in UAE. In the Croatian population the frequencies of polymorphic CYP2D6*2,*3, *4,* 5, and * 6 alleles were 4%, 2.8%, 14%, 1%, and 1.5%, respectively. The most frequently observed null allele was CYP2D6*4, which accounted for 72% of all null alleles. Among the Croatian population studied, 60% of the subjects had EM genotype, 33% were IMs, and 3% exhibited the PM genotype. Four percent exhibited the UM genotype due to amplified *CYP2D6* gene (CYP2D6*2 allele) [20]. The allele frequency of CYP2D6*4; the most common defective allele among Caucasians (25%); was 14% in Croatians [20] and only 3.5% in the Saudi population [21] and 2% in the UAE population. Two other alleles, CYP2D6*10 (47–70% in Asians) and CYP2D6*17 (25–40% in blacks), are common in certain populations resulting in diminished enzyme activity. Both were found only at low frequencies of 3.0% each in the Saudi Arabian population [21] and 3.3% and 2.5% respectively, in the UAE population.

PMs alleles (e.g.*3,*4,*4*xn*,*5,*6,*7,*8,*11 or *45) cause absent enzymatic activity and, possibly, an increased risk of adverse drug reactions even with routine therapy with *CYP2D6* substrates. CYP2D6*3,*4,*5,*6 form 93–97% of the PM phenotypes in Caucasians while other inactivating alleles form less than 1% [22], [23]. CYP2D6*4 is the most common defective allele in Caucasians with a frequency of 20–25% in both heterozygous and homozygous forms but only homozygous CYP2D6*4 is associated with the PM phenotype [24]. Heterozygous and homozygous CYP2D6*4 were found with a frequency of 9% in the Emirati population which is similar to the Palestinians (7.8%) [25] and a bit lower than the Croatians (14%) [20] but not as high as what was reported in other Caucasians [12], [24]. Three individuals (2%) were homozygous for CYP2D6*4 and were categorized as being PMs. Other defective alleles such as (*3,*5 and*6) could not be detected perhaps due to the small sample size (*n* = 151) we used for genotyping. Because of the fact that patients with CYP2D6*4 are PMs they are more than likely to develop side effects with normal doses of CYP2D*6-dependent antidepressants, particularly because most of these drugs have narrow therapeutic windows. Therefore drugs like tricyclic antidepressants (TCAs), which have a narrow therapeutic window and venalaxine are going to pose problems in these patients. Therefore lower doses to approximately half the therapeutic doses of TCA are recommended [26], [27]. The opposite is true of UMs who need higher doses of CYP2D6-dependent drugs to reach therapeutic levels.

Individuals were considered as IMs if they had one *CYP2D6* allele with a decreased enzyme activity (*9,*10,*17,*29 or *41) together with a non-functional allele (e.g. *3,*4,*4*xn*,*5,*6,*7,*8 or *11) [23] or if they had two partially functional alleles. Among the 17 variant *CYP2D6* alleles that were found in the UAE population, CYP2D6*41 allele had a high frequency of 15.2% which is higher than what was reported in other Caucasians (8.5%) [1], while closer to what was reported in Palestinians (12.7%) [25]. CYP2D6*41 occurs in 22% of Ethiopians [28]. In Asia (1%) and Africa (1–2%), it occurs in a lower frequency than in Caucasians (10%) [26]. CYP2D6*41/*null form nearly 50–70% of all Caucasian IMs [29]. In this study group 4.6% had CYP2D6*41/*null (*41/*4) genotype while 8.6% had CYP2D6*IM/*IM (e.g.*41/*41, *10/*10,*10/*41 and *17/*17). CYP2D6*10 and *17 cause a decrease in the enzyme activity. The frequency of CYP2D6*10 is relatively high in Asia (51%) and therefore nearly 25% of Asians are classified as IMs [12]. Black Africans and African-Americans have high frequencies of CYP2D6*17 allele (up to 35%) and that is the reason why the majority of them are IMs [16]. On the other hand, null alleles are much rarer in Asia and Africa than in Caucasians [1]. CYP2D6*10 and CYP2D6*17 were found in UAE at a low frequencies of 3.3% and 2% respectively, and this was expected as they occurred in even lower frequencies in some other Caucasians.

UMs are characterized by faster rate of drug metabolism caused by the presence of multiple active functional *CYP2D6* genes (at least 3 copies) on one individual allele. Consequently these subjects do not reach the therapeutic plasma levels even at normal drug doses. Alleles with multiple gene copies were found in high frequencies only in some regions such as Saudi Arabia (21%) [21] and Ethiopia (29%) [28], while in the rest of the world the frequencies do not exceed 10% [26], [30]. In UAE, the most common duplicated alleles are *1 and *2 where CYP2D6*2xn occurred in 4.3% which is close to Palestinian's frequency (4.9%) [25] and to other Caucasians (1–5%), while higher than Asians and Africans (0–2%) but still lower than Saudi Arabians and African Ethiopians (10–16%) [7]. CYP2D6*1xn occurred only in 1.6% of the Emiratis while it reached 3.7% in some Middle Eastern regions [25].

Kouhi et al. [30] determined the frequency of four *CYP2D6* alleles in Iran. He found that CYP2D6*2 that encode for an EM phenotype occurred in a frequency of 32% which when compared to what we found in UAE (12.2%) is considered high. CYP2D6*4 occurred in 12.5% of Iranians and this is somehow close to the frequency in UAE (9%). CYP2D6*10 occurred more frequently in Iran (9%) than in UAE (3.3%) while CYP2D6*17 was completely absent in Iran with a 2% frequency in UAE nationals. In addition, Iran has more PMs (4%) than UAE (2%), while they were 8% in Palestinians, 17% in Jews of Russian origin, and 0% in Jews of Yemeni and Ethiopian extraction [31].

In conclusion, *CYP2D6* gene can be considered a challenge for genotyping because its polymorphisms are numerous including not only SNPs but also gene duplication and deletion. However, our approach of direct DNA sequencing of all the coding regions and most of the intronic noncoding regions provided us with the opportunity to capture most of the variants. Our study showed that *CYP2D6* is polymorphic in the UAE population with a different distribution compared with other populations but there were some similarities especially with Palestinians. New SNPs, New alleles and atypical genotypes were observed in the UAE study group (Table 1 and 2). Expanding the number of participants and further studies are anticipated to enrich the *CYP2D6* nomenclature database and increase our knowledge of CYP2D6 among Emiratis. In addition, we believe that family based studies would be helpful in determining the full haplotypes in this population. MRs especially for the new alleles should be measured to verify the corresponding phenotype.

## Materials and Methods

### Subjects blood sampling

#### Ethics Statement

This study was approved by Al-Ain District Human Research Ethics and the Faculty of Medicine and Health Sciences, UAE University Committee (07/148) and informed consent was obtained from all subjects.

Blood samples were collected from 151 Emiratis: 101 of them were unrelated UAE nationals who suffered from endogenous depression and were being treated with antidepressants at Al Ain Hospital inpatient and outpatient psychiatry clinics as well as Tawam Hospital outpatient clinic. The other 50 individuals were blood donors randomly selected from healthy population.

### Extraction of DNA

DNA was extracted from peripheral leukocytes using a whole-blood Qiagen extraction kit (Flexigene DNA isolation kit). The isolated genomic DNA samples were kept in sterile plastic vials at 4°C until analysis or stored at −20°.

### PCR amplification of the *CYP2D* gene and DNA sequencing

Primer design and PCR amplification was achieved under standard conditions described by Masimirembwa et al. (1996) [8]. The oligonucleotides used for PCR are listed in table S1. DNA was sequenced using the BigDye Terminator kit v3.1with a 3130x/genetic analyzer system (Applied Biosystems) following ExoSAP-IT® (USB Corporation) treatment of the PCR products. Cycle sequencing was performed under standard conditions recommended by the manufacturers (Applied Biosystems Inc.). Oligonucleotides used for sequencing are listed in table S2.

### Analysis of sequencing data

For each patient the produced sequences were aligned with the original *CYP2D6* sequence (NM_000106.4) using ClustalW2 (http://www.ebi.ac.uk/Tools/msa/clustalw2/) to detect the presence of SNPs or mutations as well as to define each patient's genotype. Expasy Translate tool (http://us.expasy.org/tools/dna.html) was used to translate DNA sequences into their protein sequences to examine the influence of the DNA changes at the protein level and thus the enzymatic activity.

### TaqMan RT-PCR

*CYP2D6 gene* copy number was determined using TaqMan® real-time quantitative PCR (ABI 7900 Fast Real-Time PCR system; Applied Biosystems). Real time data were collected by the SDS 2.1 software, and data were analyzed using the relative quantification (ΔΔC_T_) method. Three calibrators (containing one, two or three *CYP2D6* gene copies DNA samples from Coriell Inc., http://ccr.coriell.org) were included in each run for the accuracy of the results and each sample was normalized to RNaseP to produce ΔC_T_. All samples were then normalized to the calibrator (NA17120) sample to determine ΔΔC_T_. Relative quantity (RQ) values were then calculated and multiplied by 2 to produce *CYP2D6* gene copy number.

### Statistical analysis

The statistical analysis was conducted by SPSS 17.0 (for windows) to calculate frequencies. The level of significant of *P* value was set at less than 0.05.

## Supporting Information

Table S1 **Primers for PCR amplification of** ***CYP2D6*** **gene (exons 1–9).**
(DOCX)

Table S2 **Primers for** ***CYP2D6*** **sequencing.**
(DOCX)
