# Supplementary material for: Identification of New Alleles and the Determination of Alleles and Genotypes Frequencies at the CYP2D6 Gene in Emiratis
Source: PLoS One. 2011 Dec 22;6(12):e28943. doi: 10.1371/journal.pone.0028943 (PMC3245235; doi:10.1371/journal.pone.0028943)
Supplement: Table S1 — Primers for PCR amplification of CYP2D6 gene (exons 1–9). (DOCX) [file pone.0028943.s001.docx]

**Supplementary Table 1.** Primers for PCR amplification of *CYP2D6* gene *(*exons 1-9)

| **Exon** | **Primer sequence** | **Amplicon size** |
| --- | --- | --- |
| Exon 1 and 2 | Forward: 5΄- ACCAGGCCCCTCCACCGG-3΄  Reverse: 5΄- CTCTCTGCCCAGCTCGG-3΄ | 1414 |
| Exon 3 and 4 | Forward: 5΄-ATTTCCCAGCTGGAATCC-3΄  Reverse: 5΄-GAGACTCCTCGGTCTCTC-3΄ | 740 |
| Exon 5 and 6 | Forward: 5΄-GCCTGAGACTTGTCCAGG-3΄  Reverse: 5΄-CCGGCCCTGACACTCCTTCT-3΄ | 738 |
| Exon 7,8 and 9 | Forward: 5΄-CAACATAGGAGGCAAGAAG-3΄  Reverse: 5΄-ATATAGCTCCCTGACGCC-3΄ | 1639 |
