# Supplementary material for: Identification of New Alleles and the Determination of Alleles and Genotypes Frequencies at the CYP2D6 Gene in Emiratis
Source: PLoS One. 2011 Dec 22;6(12):e28943. doi: 10.1371/journal.pone.0028943 (PMC3245235; doi:10.1371/journal.pone.0028943)
Supplement: Table S2 — Primers for CYP2D6 sequencing. (DOCX) [file pone.0028943.s002.docx]

**Supplementary Table 2.** Primers for *CYP2D6* sequencing

| **Exon** | **Primer Sequence** |
| --- | --- |
| Exon 1 | Forward : 5΄- ACCAGGCCCCTCCACCGG-3΄ |
| Exon 2 | Forward : 5 ´- GTCTCCTCCTTCCACCTGCT- 3΄ |
| Exon 3 and 4 | Forward : 5 ´- ATAGGGTTGGAGTGGGTGGT-3΄ |
| Exon 5 and 6 | Reverse : 5΄ -CCGGCCCTGACACTCCTTCT-3΄ |
| Exon 7 | Reverse : 5´-TGTGGTGGCATTGAGGACTA-3´ |
| Exon 8 and 9 | Forward : 5´-GTCGGAGAGGGTACTGTGGA-3´ |
